# The Johns Hopkins Hospital Reports

**Published:** 1903-01

**Authors:** 


THE JOHNS HOPKINS HOSPITAL REPORTS. Volume X., Nos. 3, 4 and 5. Baltimore: The Johns Hopkins Press. 1902.

The investigation comprising this report are of a pathological nature and worthy of careful study and consideration.

Dr. Dorothy M. Reed, of the hospital, contributes an interesting article on the pathological changes in Hodgkin's disease with especial reference to its relation to tuberculosis. The author feels that although the pathological agent is as yet undiscovered, it has no direct relation with tuberculosis, and that a microscopic examination is sufficient for the diagnosis. Dr. Thomas Futcher, M.B., contributes an article on diabetes insipidus with a report of five cases; Drs. W. T. Howard, Jr., and R. G. Perkins, of Cleveland, O., contribute a very interesting investigation on the origin and occurrence of cases with eosinophile granulations in normal and pathological tissues. Dr. Frank W. Lynch presents a paper on placental transmission, with report of a case during typhoid fever. W. C. K.

